# Supplementary figures and images for: Quantifying acyl-chain diversity in isobaric compound lipids containing monomethyl branched-chain fatty acids
Source: J Lipid Res. 2024 Oct 26;65(12):100677. doi: 10.1016/j.jlr.2024.100677 (PMC11621494; doi:10.1016/j.jlr.2024.100677)

### DB-35MS column

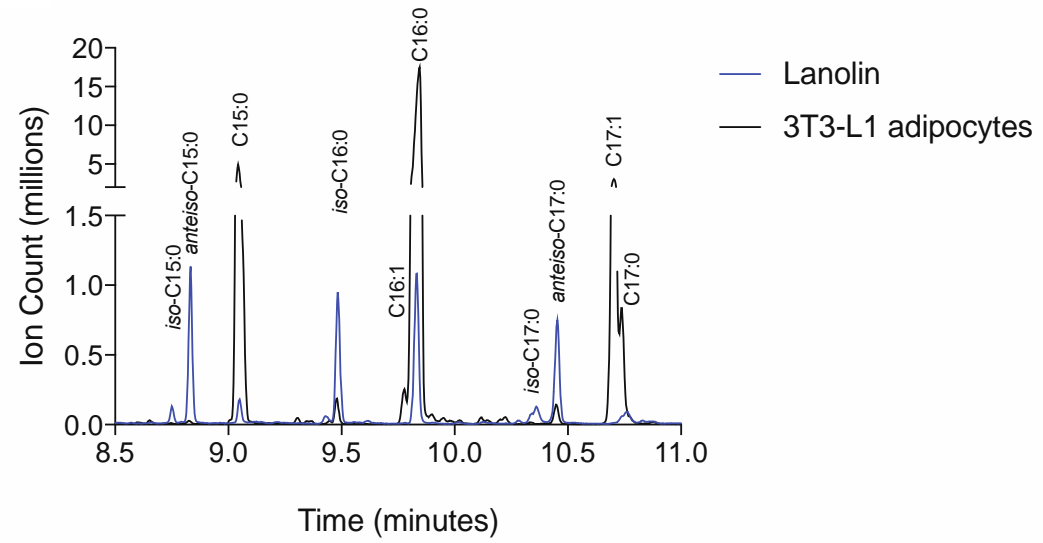

Supplement: Supplemental Figure 1 — Branched- and straight-chain fatty acids are chromatographically resolvable on GC-MS. Separation of free monomethyl BCFAs and straight chain fatty acids as fatty acid methyl esters in lanolin extract and 3T3-L1 adipocytes using via GC-MS using a DB-35MS column. [file mmc1.pdf]

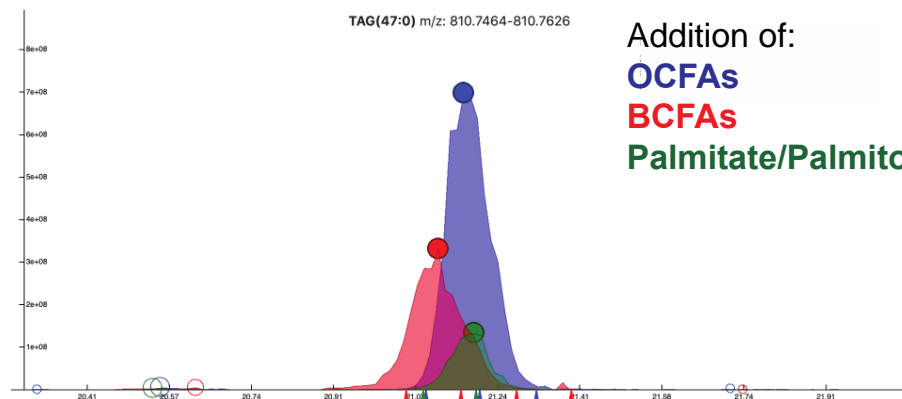

C18 column

Supplement: Supplemental Figure 2 — Experimental conditions from Figure 1, ran on Kinetex C18 column (1.7 μm, 250 × 2.1 mm, Phenomenex). [file mmc2.pdf]

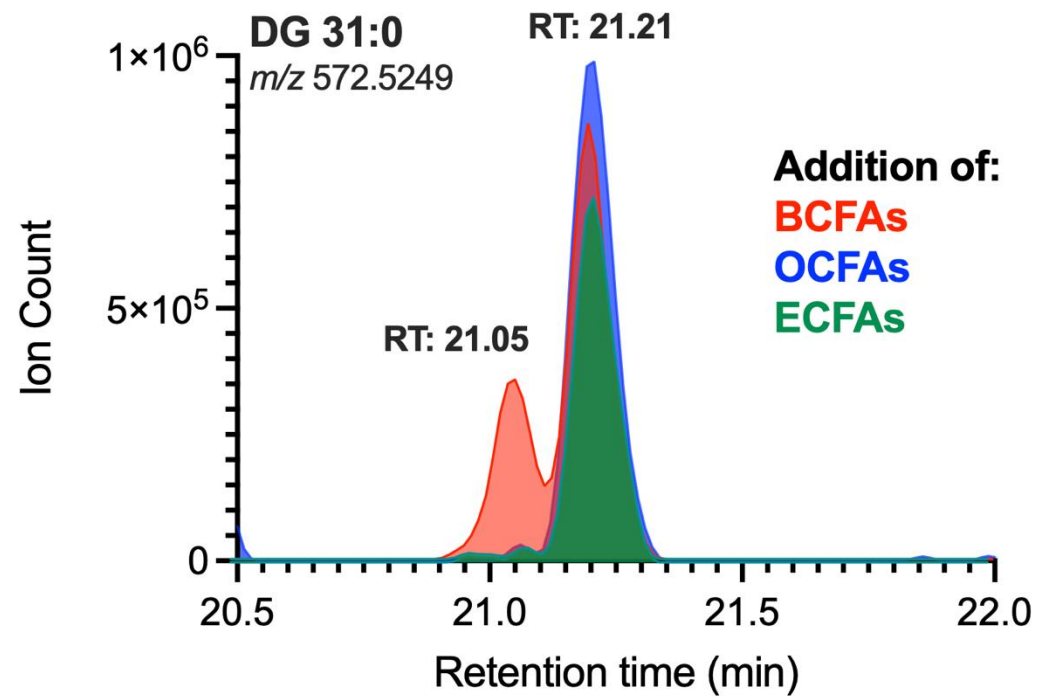

Supplement: Supplemental Figure 3 — Overlay of extracted ion chromatograms of DG 31:0 (m/z 572.5249 ± 5 ppm, corresponding to the [M+NH4]+ adduct ion) from each experimental condition supplementing 3T3-L1 adipocytes with either ECFAs (green), OCFAs (blue), or BCFAs (red) using UHPLC-MS equipped with an Accucore C30 (2.6 μm, 250 × 2.1 mm, Thermo). [file mmc3.pdf]

**A**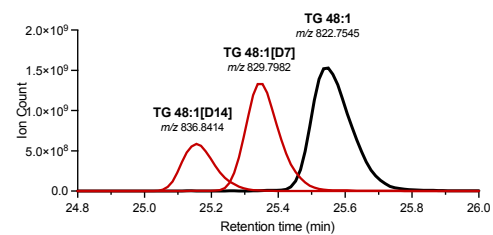**B**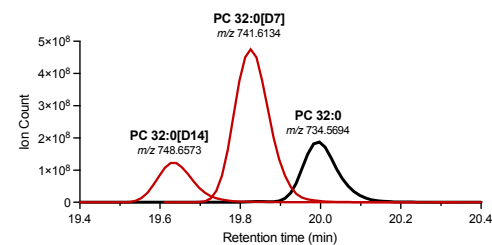**C**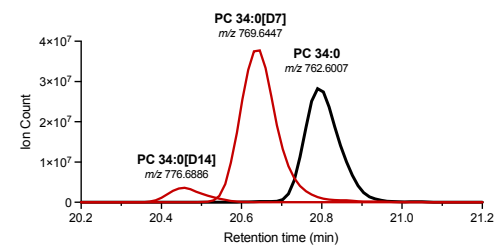**D**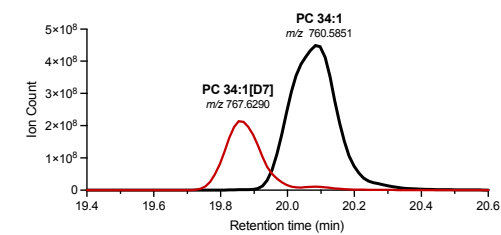**E**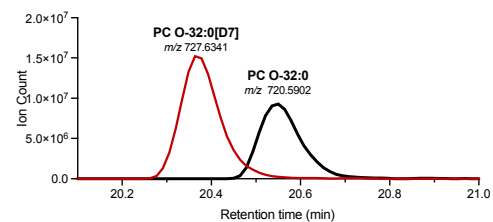**F**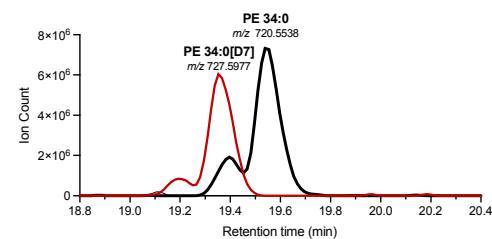**G**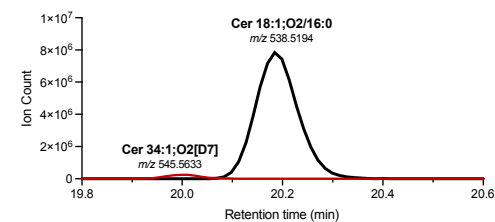**H**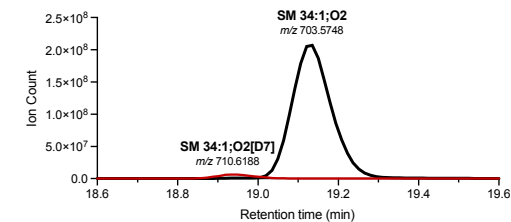**I**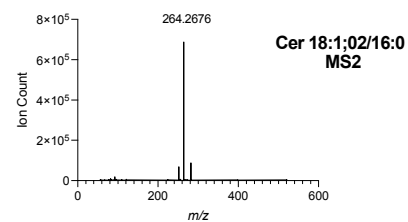

Supplement: Supplemental Figure 4 — A–H: Extraction ion chromatograms illustrating iso-FA 16:0[D7] integration across different complex lipids. Retention time shifts highlighted with the incorporation of each BCFA acyl chain. Respective extracted ion m/z displayed in each figure; ion adduct extracted shown below for each class of lipids. TG, triacylglycerol; PC, phosphatidylcholine; PE, phosphatidylethanolamine; Cer, ceramide; SM, sphingomyelin. A: TG 48:1 [M+NH4]+. B: PC 32:0 [M+H]+. C: PC 34:0 [M+H]+. D. PC 34:1 [M+H]+. E: PC O-32:0 [M+H]+. F: PE 34:0 [M+H]+. G: Cer 18:1;O2/16:0 [M+H]+. H: SM 34:1;O2 [M+H]+. I. MS2 spectra of ceramide from Supplemental Fig. S4G shows a characteristic m/z of 264.2676 representing a fragment product of SPB 18:1; O2. [file mmc4.pdf]

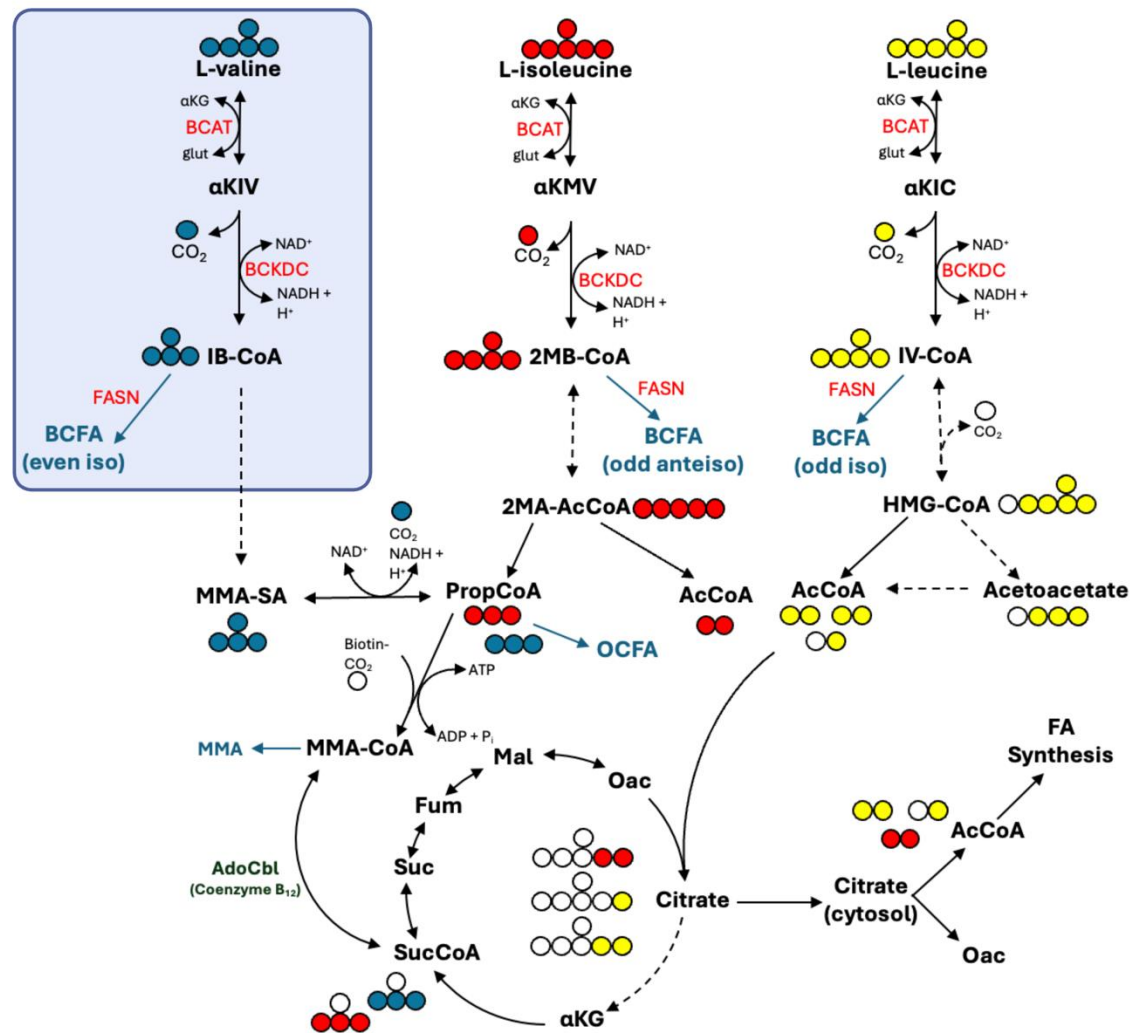

Supplement: Supplemental Figure 5 — Metabolic map depicting the catabolism of the branched-chain amino acids (BCAA) valine, isoleucine, and leucine. Oxidation of [U-13C5]valine leads to BCFA synthesis (highlighted in the blue box), OCFA synthesis, and TCA cycle incorporation. Key enzymes for BCFA biosynthesis are highlighted in red text. BCAT, branched-chain amino acid aminotransferase; BCKDC/Bckdha, branched-chain a-ketoacid dehydrogenase complex; FASN, fatty acid synthase. [file mmc5.pdf]
